# Reply to Darlow and Gray: Censorship is exclusion

**DOI:** 10.1073/pnas.2404156121

**Published:** 2024-05-13

**Authors:** Cory J. Clark, Musa al-Gharbi, Roy F. Baumeister, April Bleske-Rechek, David Buss, Stephen Ceci, Joseph Forgas, Komi Frey, David C. Geary, Glenn Geher, Marco Del Giudice, Lee S. Jussim, Anna I. Krylov, Chris Martin, Geoffrey Miller, Pamela Paresky, Catherine Salmon, Steve Stewart-Williams, Anne E. Wilson, Wendy Williams, Bo M. Winegard, William von Hippel

**Affiliations:** ^a^School of Arts and Sciences, University of Pennsylvania, Philadelphia, PA 9104; ^b^The Wharton School, University of Pennsylvania, Philadelphia, PA 9104; ^c^School of Communication and Journalism, Stony Brook University, Long Island, NY 11794; ^d^School of Psychology, University of Queensland, St Lucia, QLD 4072, Australia; ^e^Department of Psychology, University of Wisconsin-Eau Claire, Eau Claire, WI 54702; ^f^Department of Psychology, University of Texas at Austin, Austin, TX 78731; ^g^Department of Psychology, Cornell University, Ithaca, NY 14853; ^h^School of Psychology, The University of New South Wales, Sydney, NSW 2052, Australia; ^i^Research Department, Foundation for Individual Rights and Expression, Philadelphia, PA 19106; ^j^Department of Psychological Sciences, University of Missouri, Columbia, MO 56211; ^k^Department of Psychology, State University of New York at New Paltz, New Paltz, NY 12561; ^l^Department of Life Sciences, University of Trieste, Trieste 34128, Italy; ^m^Department of Psychology, Rutgers University, Piscataway, NJ 08854; ^n^Department of Chemistry, University of Southern California, Los Angeles, CA 90089; ^o^Psychology Department, Oglethorpe University, Brookhaven, GA 30319; ^p^Department of Psychology, University of New Mexico, Albuquerque, NM 87131; ^q^Network Contagion Research Institute, Princeton, NJ 08540; ^r^Department of Psychology, University of Redlands, Redlands, CA 92373; ^s^School of Psychology, University of Nottingham Malaysia, Selangor Darul Ehsan, Semenyih 43500, Malaysia; ^t^Psychology Department, Wilfrid Laurier University, Waterloo, ON N2L3C5, Canada; ^u^ Independent researcher; ^v^Research with Impact, Brisbane, QLD 4069, Australia

We thank Darlow and Gray (henceforth D&G) (1) for raising possible misconceptions regarding our paper on scientific censorship (2). First, D&G conflate explanation with blame; to explain is not to blame, and the concept of blame is irrelevant to our paper. Next, D&G write, “the authors suggest that increased participation of women and people from diverse backgrounds in academia increases censorship….” While we proposed harm-aversion as one explanation for women supporting censorship more than men, we made no claims about people from diverse backgrounds, nor are we aware of relevant data. D&G then combine ethical concerns regarding the treatment of participants in research with harm concerns regarding the dissemination of science. Censorship pertains only to the latter.

D&G note that one study we cited (3) had a very low response rate, which led Gray (the second author of D&G) (1) to file an official complaint regarding the credibility of the findings. We agree that with such a low response rate, the report may over- or underrepresent true rates of self-censorship among New Zealand academics and that more research is needed.

D&G state, “None of the solutions proposed address underlying problems that give rise to prosocial concerns.” To the contrary, greater transparency and academic audits can help identify any nonscientific basis for editorial decisions or double standards, including discrimination. Audit studies are a rigorous method for identifying discrimination (4) and safeguards against censorship protect minority perspectives.

D&G suggest that censorship is necessary to prevent harm but that it would be unnecessary if vulnerable groups were involved in scholarship affecting their communities. As noted in our paper, there are no data on the costs and benefits of censorship (or alternative harm-prevention solutions) upon which to base such judgments. Additionally, involving community members in research may not eliminate harm-avoidant censorship. Roland Fryer, a Black scholar from an impoverished community, overcame soft-censorship to publish his findings regarding (a lack of) racial differences in the victims of police officer shootings, (5) reporting that he “had colleagues take me to the side and say, ‘Don’t publish this. You’ll ruin your career.’”

D&G argue that “All research pertaining to specific communities should involve active participation of those communities.” Assuming D&G refer to involvement as coinvestigators rather than as research participants; this demand is impractical and unnecessarily restrictive. Just as insiders have provided insights, so have outsiders (e.g., when left-leaning scholars study right-leaning ideologies).

D&G appear to agree with our main point that scientific censorship is often driven by prosocially motivated scientists. They suggest this censorship is warranted, whereas we call for caution. As described in our paper, many sources of data could enable metascientific investigation of the frequency, costs, and benefits of this form of censorship, but in the absence of such investigation, we are “left to quarrel based on competing values, assumptions, and intuitions.” D&G’s letter exemplifies just such an outcome.
